# Identification of biomarkers in Parkinson’s disease by comparative transcriptome analysis and WGCNA highlights the role of oligodendrocyte precursor cells

**DOI:** 10.3389/fnagi.2024.1485722

**Published:** 2024-11-20

**Authors:** Fa-Li Zhang, Ai-Ying Li, Yi-Lin Niu, Kai Zhang, Ming-Hui Zhao, Jiao-Jiao Huang, Wei Shen

**Affiliations:** ^1^College of Life Sciences, Qingdao Agricultural University, Qingdao, China; ^2^College of Animal Science and Veterinary Medicine, Shandong Agricultural University, Tai'an, China

**Keywords:** Parkinson, OPCs, oligodendrocyte, WGCNA, biomarker

## Abstract

**Background:**

Parkinson’s disease (PD) is an age-related neurodegenerative disease characterized by the death of dopamine neurons in the substantia nigra. A large number of studies have focused on dopamine neurons themselves, but so far, the pathogenesis of PD has not been fully elucidated.

**Results:**

Here, we explored the significance of oligodendrocyte precursor cells (OPCs)/oligodendrocytes in the pathogenesis of PD using a bioinformatic approach. WGCNA analysis suggested that abnormal development of oligodendrocytes may play a key role in early PD. To verify the transcriptional dynamics of OPCs/oligodendrocytes, we performed differential analysis, cell trajectory construction, cell communication analysis and hdWGCNA analysis using single-cell data from PD patients. Interestingly, the results indicated that there was overlap between hub genes and differentially expressed genes (DEGs) in OPCs not in oligodendrocytes, suggesting that OPCs may be more sensitive to PD drivers. Then, we used ROC binary analysis model to identify five potential biomarkers, including AGPAT4, DNM3, PPP1R12B, PPP2R2B, and LINC00486.

**Conclusion:**

In conclusion, our work highlights the potential role of OPCs in driving PD.

## 1 Introduction

Parkinson’s disease (PD) is the second most common neurodegenerative disease, the main pathological feature of which is the degeneration and death of dopaminergic neurons in the substantia nigra compacta of the midbrain (Coukos and Krainc, 2024; Ben-Shlomo et al., 2024). Causes of dopamine neuron cell damage include but are not limited to, *α*-Synuclein aggregation and the presence of Lewy bodies, oxidative stress triggering mitochondrial dysfunction, and abnormal autophagy [see review (Poewe et al., 2017; Dexter and Jenner, 2013; Dong-Chen et al., 2023) for details]. Despite extensive work in decoding the molecular mechanisms driving PD, the heterogeneous etiology of PD has not been fully answered to date (Surguchov, 2022). Indeed, most research focuses on dopamine neurons themselves while ignoring other cell types, such as glial cells (Guo et al., 2018; Surmeier, 2018; Xiao et al., 2023). Currently, with the rise of single-cell sequencing technology, some work has attempted to uncover the progression of PD from the perspective of cell communication and cellular heterogeneity at the single-cell level (Huang et al., 2022; Smajić et al., 2022). Emerging clues suggest that drivers of PD progression may lie outside dopamine neurons or between dopamine neurons and other cells (Huang et al., 2022; Drobny et al., 2021). Hence, decoding the transcriptional signatures of cells other than dopamine neurons is capable of providing valuable information on the mechanisms of PD.

There is a consensus that the correct execution of brain functions requires integrity in which functionally distinct neurons and non-neuronal cells interact in a coordinated and tightly regulated manner. Neuronal/oligodendrocyte precursor cells (OPCs) (Maldonado and Angulo, 2015; Xiao and Czopka, 2023), neuronal/oligodendrocytes (Mitew et al., 2018; Azevedo et al., 2022) have been characterized, but it is not enough. OPCs are also called NG2-glial cells or O2A cells. Their main function is to serve as precursors of oligodendrocytes (Hayashi and Suzuki, 2019). Functionally, OPCs give rise to oligodendrocytes, which then wrap around axons and form myelin to provide electrical insulation (Nishiyama et al., 2021; Mi et al., 2009). Additionally, the most intriguing facet of OPCs is the fact that they represent the only type of glial cells that receives direct synaptic inputs from neurons and exhibits neuronal-like long-term potentiation (LTP) at excitatory synapses (Zhang et al., 2021). And it is widely accepted that OPCs participate in neuronal circuitries in health and disease (Nagai et al., 2019). Furthermore, deficiency of NG2 glia contributes to neuroinflammation and nigral dopaminergic neuron loss in MPTP-induced mouse PD model (Zhang et al., 2019). A recent study reports that transcriptome changes in oligodendrocytes and OPCs can predict clinical outcomes in PD. This work identified a unique subtype of OPCs that showed predictive power for movement disorders and was significantly increased in PD (Dehestani et al., 2023). The above provides an exciting perspective that genetic disturbances in OPCs/oligodendrocytes may serve as predictive targets for PD. Identification of PD biomarkers that can be used in clinical diagnosis and research, such as extensive molecular genetic studies, is crucial; however, these biomarkers remain to be explored, especially in non-neuronal cells. Weighted gene co-expression network analysis (WGCNA) has been extensively used in different fields, such as PD (Jin et al., 2020), reproductive development (Zhang et al., 2022; Zhang et al., 2023b), to explore the association between gene networks and phenotypes of interest, as well as the hub genes in the network, and ultimately identifies biomarkers. Recently, an exciting work has provided WGCNA on single-cell datasets, called hdWGCNA, which enables researchers to perform more efficient identification of single-cell data (Morabito et al., 2023).

Unfortunately, early PD studies in humans are almost impossible due to various limitations. Therefore, it is very important to use animal models to study the regulatory mechanism of early PD. In this study, a rat PD model dataset was selected for WGCNA analysis to obtain the key genes that regulate early PD. This model can reshape the main case features of PD in rats, such as the formation of Lewy bodies (Hentrich et al., 2020). The results repute that PD progression may be associated with oligodendrocyte transcriptional abnormalities. Further, we performed hdWGCNA analysis on OPCs/oligodendrocytes/neuronal cells of two human brain regions in single-cell transcriptomes. Interestingly, our results highlight that the early stages of PD may be driven by OPCs rather than oligodendrocytes.

## 2 Methods and materials

### 2.1 Dataset correction and preprocessing

To investigate the progression mechanism of PD and perform WGCNA, we first collected bulk RNA-seq data (GSE150646) from an *SNCA* overexpression rat model, including 20 samples (Hentrich et al., 2020). Quality control of single-cell samples is based on the number of genes detected, the number of total RNA molecules detected, and the percentage of mitochondria or ribosomes from each sample. Since they come from different experiments, we adopt different data filtering methods. Expressly, for GSE140231, this study first excludes the data of two cases of cerebral amyloid angiopathy and it only includes four cortex single cell data (Agarwal et al., 2020), and the data filtering threshold is 10,000 > nCounts_RNA > 1,000, nFeature >200, and the percentage of mitochondrial genes <10%. For GSE157783, all samples were included in the study and the data filtering threshold is nCounts_RNA > 1,000, nFeature >200, and the percentage of ribosomes genes <0.05% (Smajić et al., 2022). Moreover, we used a bulk RNA-seq dataset (GSE205450) of one human brain region for validation (Irmady et al., 2023).

This is an observational study. The Ethics Committee of Qingdao Agricultural University has confirmed that no ethical approval is required.

### 2.2 The workflow of scRNA-seq

After obtaining high-quality single-cell data through quality control, the data were processed using the Seurat (R software package v4.4.0) (Hao et al., 2021). The two datasets were then merged, and dimension reduction was performed using the canonical correlation analysis algorithm. The dimensionality reduction and clustering results are visualized by the uniform manifold approximate projection (UMAP) method at a resolution of 0.3 and a dimension of 10 via plot1cell (R software package v0.0.0.9000) or Seurat (R software package v4.4.0) (Hao et al., 2021; Wu et al., 2022). Next, cluster annotation using classic marker genes, Oligodendrocytes [*MOBP*, *MOG* (Montague et al., 2006; Juryńczyk et al., 2019)], OPCs [*VCAN* (van Bruggen et al., 2017)], Astrocyte [*AQP4, GFAP* (Ikeshima-Kataoka, 2016; Jurga et al., 2021)], Neuronal [*GAD1, GAD2* (Kodama et al., 2012)], Microglia [*CD74* (Hwang et al., 2017)], Endothelial [*EGFL7, CLDN5* (Jang et al., 2011)] and Ependymal [*FOXJ1* (Shah et al., 2018)].

### 2.3 Identification of differentially expressed genes (DEGs)

For bulk RNA-seq data, the DESeq2 (R software package v1.42.1) was used for the DEGs detection, and the input was the gene counts matrix (Love et al., 2014). Only genes satisfying *padj* < 0.05 & |log2FoldChange| > 0.5 were considered DEGs. For scRNA-seq data, *FindMarkers()* function of the Seurat was used for DEGs detection (Hao et al., 2021), and the threshold was set to logfc.threshold >0.1, min.pct > 0.1 and *pvalue < 0.05.* Unless otherwise specified, the default algorithms and parameters of the software were used.

### 2.4 Gene functional enrichment analysis

For functional exploration of the gene sets, including DEGs, hub genes and candidate genes, functional enrichment analysis, including Gene Ontology (GO) and Kyoto Encyclopedia of Genes and Genomes (KEGG), was performed. The clusterProfiler (R software package v4.10.1) and metascape (v3.5.20240101^1^) were used for gene functional enrichment analysis (Wu et al., 2021; Zhou et al., 2019). The significance level of *padj* < 0.05 was considered as the cut-off threshold.

### 2.5 Weighted gene co-expression network analysis (WGCNA)

For bulk RNA-seq data, the WGCNA (R software package v1.72–5) was used for WGCNA, and the input was the gene counts matrix. First, missing values and outlier samples were checked (Langfelder and Horvath, 2008). The *hclust()* function was used to check outlier samples, and samples with obvious outliers were excluded from the analysis. Next, the *pickSoftThreshold()* function was used to calculate and pick a suitable soft threshold. The *blockwiseModules()* function was used to construct overexpression networks and module partitioning in one step, and the parameters we set are *power = sft$powerEstimate, maxBlockSize = 6,000, TOMType = “unsigned,” minModuleSize = 300, mergeCutHeight = 0.3 and deepSplit = 2*. Subsequently, the *labeledHeatmap()* function was used to visualize the relationship between modules and phenotypes. Note that since the genes in the gray modules did not participate in the clustering of any module, they were not included in the subsequent analysis. The module membership (MM) and gene significance (GS) algorithm was used to mine hub genes of WGCNA in bulk RNA-seq, which are often closely related to traits (Zhang et al., 2022; Zhang et al., 2023b). The cut-off threshold of hub genes was MM > 0.6 and GS > 0.4.

For scRNA-seq data, the hdWGCNA (R software package v0.3.03) was used for WGCNA, and the input was the integrated Seurat object (Morabito et al., 2023). First, we filtered genes. We only included genes expressed in more than 5% of cells as subsequent genes. Merging multiple cell expression patterns (KNN algorithm) into one metacell can avoid the sparsity of single-cell data. Next, three cell types, Oligodendrocytes, Neuronal, and OPCs, were selected for WGCNA analysis. The *TestSoftPowers()* function was used for appropriate soft threshold screening and visualization. The *ConstructNetwork()* function was used for scale-free network construction and module division. The *GetHubGenes()* function obtained hub genes in different modules.

### 2.6 Construction of pseudo-time trajectories

To investigate the transcriptional dynamics of genes in oligodendrocytes and OPCs during PD progression, we performed pseudo-time trajectories analysis by monocle (R software package v2.24.0), which constructs cell lineage development based on the changes in gene expression levels of different cell subsets over time (Trapnell et al., 2014). The *reduceDimension()* function is used to determine the trajectory, and the *orderCells()* function is used to sort cells. Since the algorithm trajectory does not conform to the actual biological law, we manually specified root_state = 2 in this step. The BEAM statistical analysis model was used to calculate cell fate trajectories before and after key fate nodes, using unsupervised clustering genes as markers.

### 2.7 Analysis of cell–cell communications

To characterize the differences in signal transduction pathways between normal physiological conditions and PD, the CellChat (R software package v1.6.1) algorithm was used to analyze intercellular communication at the single-cell level (Jin et al., 2021). The Cell Communication Database uses CellChatDB.human as a reference. The global cell–cell communication network between normal and PD group was quantitatively and comparatively analyzed using the *compareInteractions()* function. The *netVisual_bubble()* function was used to visualize the differences in cellular communication between OPCs-neuronal and oligodendrocytes-neuronal.

### 2.8 Protein–protein interaction (PPI) network analysis

Proteins interact with each other to form complex interaction networks to regulate various aspects of life processes, such as gene expression regulation, cell cycle regulation, etc. (Tomkins and Manzoni, 2021). The candidate genes were used as inputs for PPI construction using the String online database.^2^ The default parameters were run, and we removed node proteins with no interactions during visualization.

### 2.9 Construction of ROC binary analysis model

ROC curve analysis was used to evaluate the diagnostic value of candidate genes for PD and to obtain the final biomarker. The pROC (R software package v1.18.5) was used to calculate ROC curve and visualization. Data from separate datasets (GSE205450) were used for input.

## 3 Results

### 3.1 *SNCA* overexpression causes gene expression disorder in rat brain

PD is a neurodegenerative disease that is closely related to age (Coukos and Krainc, 2024). However, the mechanism of PD progression has not been fully elucidated. Here, we first analyzed the data from the *SNCA* overexpression rat model and showed that *SNCA* overexpression could disrupt rat frontocortical gene transcription at an early stage. A total of 2,072 DEGs were obtained, including 1,433 up-regulated genes and 639 down-regulated genes (Supplementary Figure S1A). GO enrichment analysis showed that items related to learning and cognition were significantly enriched, such as “learning or memory,” “cognition,” “learning” (Supplementary Figure S1B). KEGG pathway results suggested that “Neuroactive ligand-receptor interaction” was the most enriched pathway (Supplementary Figure S1C). Interestingly, in the late *SNCA* overexpression stage (named PD_old group), only 211 DEGs were detected (Supplementary Figure S1D). The GO terms results showed that they were related to “glycosylceramide metabolic process,” “ensheathment of neurons,” and so on (Supplementary Figure S1E), and no KEGG pathway was enriched.

### 3.2 WGCNA identifies critical modules involved in PD progression

**Figure 1:**
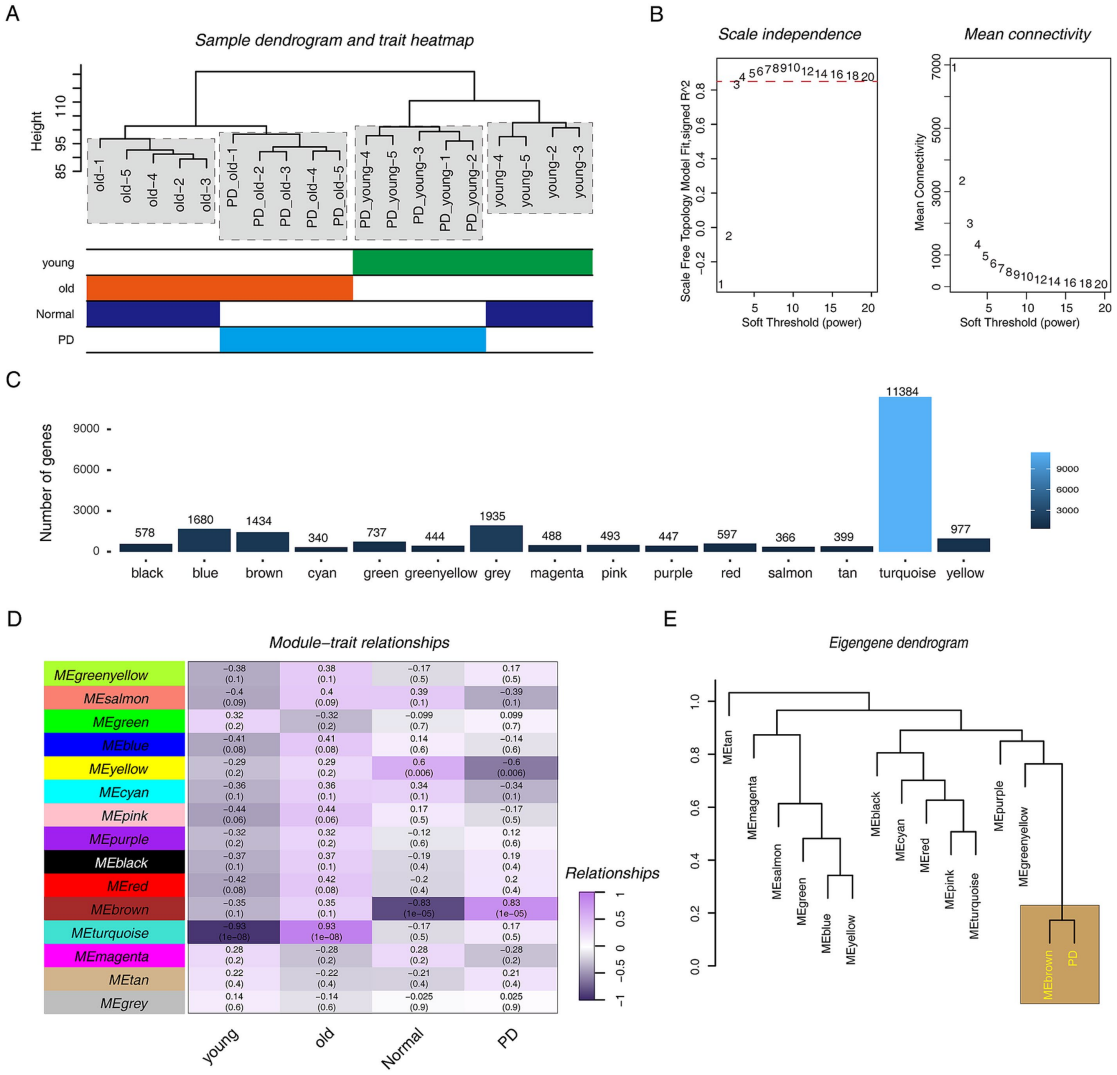
WGCNA analysis in PD rat models. (A) The hierarchical clustering tree shows the discreteness of different samples, and the different color blocks below represent different phenotypes. (B) Scale-free network topology analysis with different soft threshold powers in scale-free networks. (C) The bar graph shows the number of genes contained in different modules. (D) The heat map shows the correlation between different modules and phenotypes. Purple represents positive correlation, black represents negative correlation, numbers represent correlation coefficients, and numbers in brackets represent *p-*value. (E) The hierarchical clustering diagram shows the relationship between PD progression and various modules.

To gain insight into the pathogenesis of PD, especially in its early stages, we used the WGCNA approach. The hierarchical clustering tree showed that one sample had significant outlier performance, so we removed it (Figure 1A; Supplementary Figure S2A). We constructed a scale-free co-expression network for the remaining samples. First, we performed soft threshold selection. The results showed that it performed well and met the goal of building a scale-free network when *β* = 4 (Figure 1B). Fifteen modules were obtained, among which the turquoise module had the largest number of genes, the cyan module had the least number of genes, and the grey module contained no meaningless genes (Figure 1C). We next attempted to explore the relationship between these modules and PD progression, and the results indicated that the brown module was obviously positively correlated with PD progression (*r* = 0.83, *p* = 1e-05) and clearly negatively correlated with the normal phenotype (*r* = −0.83, *p* = 1e-05) (Figure 1D). Furthermore, we constructed a clustering matrix of PD phenotypes and modules, and the results showed that the brown module was closely associated with PD (Figure 1E).

### 3.3 Key module annotation suggests that PD is associated with oligodendrocyte abnormalities

**Figure 2:**
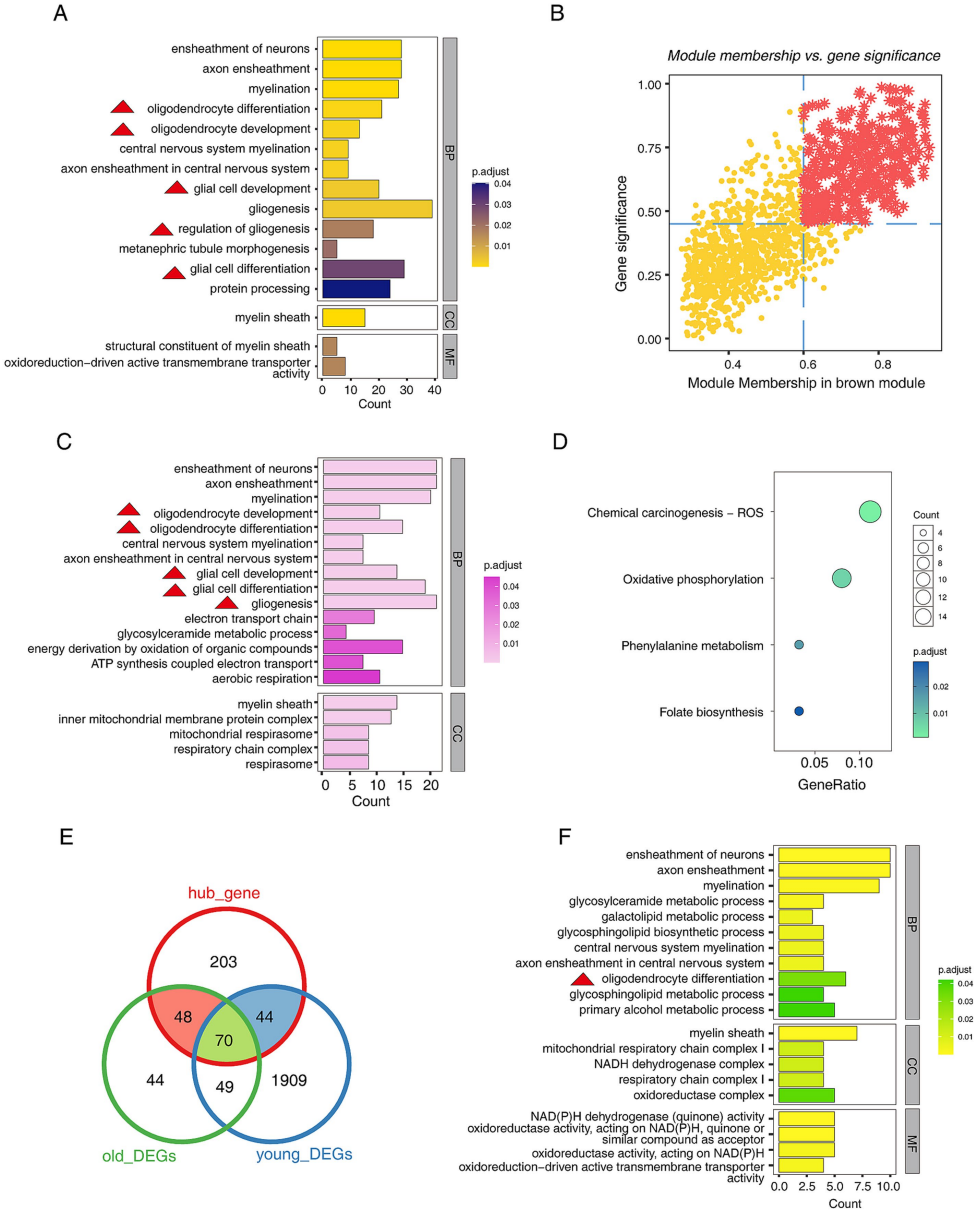
Functional exploration of genes in the key modules of WGCNA. (A) The bar plot shows the top 20 GO functional annotations of genes in the brown module, including biological Process (BP), cellular component (CC), and molecular function (MF). (B) The scatter plot shows the hub genes screening by MM&GS algorithm, and the red star points are hub genes. (C) The bar plot shows the top 20 GO functional annotations of hub genes, including BP and CC. (D) The dot plot shows the KEGG pathways of hub genes. (E) The Venn diagram shows the relationship between hub genes and DEGs. (F) The bar plot shows the top 20 GO functional annotations of shared genes between hub genes and DEGs, including BP, CC, and MF. Of note, the red triangles appearing in the functional annotations represent entries related to glial cell development.

After identifying the key modules, we explored the functions of the genes within the brown modules. The GO annotation results showed that the genes in the module were involved in “oligodendrocyte differentiation,” “oligodendrocyte development,” “glial cell development,” “regulation of gliogenesis,” etc. Moreover, “oxidoreduction-driven active transmembrane transporter activity” was also enriched (Figure 2A). This result suggests that abnormalities in oligodendrocytes may drive the progression of PD. Next, the MM&GS algorithm identified the hub gene in the brown module (Figure 2B), and functional annotation of hub genes was also performed. The GO annotation results showed that the hub genes were related to “oligodendrocyte differentiation,” “oligodendrocyte development,” “glial cell development,” “ATP synthesis coupled electron transport,” etc. (Figure 2C). The KEGG pathway results showed that the hub genes were involved in “oxidative phosphorylation” (Figure 2D). This result suggests that mitochondrial dysfunction is also involved in the progression of PD. Subsequently, we compared the relationship between hub genes and DEGs. The results showed that hub genes had 162 overlapping DEGs in the two groups (Figure 2E). The GO annotation of 162 genes indicated that they were associated with “oligodendrocyte differentiation,” “mitochondrial respiratory chain complex I,” etc. (Figure 2F). The above results strongly suggested that oligodendrocyte abnormalities are significant for the progression of PD.

### 3.4 scRNA-seq altas of human brain in normal and PD

**Figure 3:**
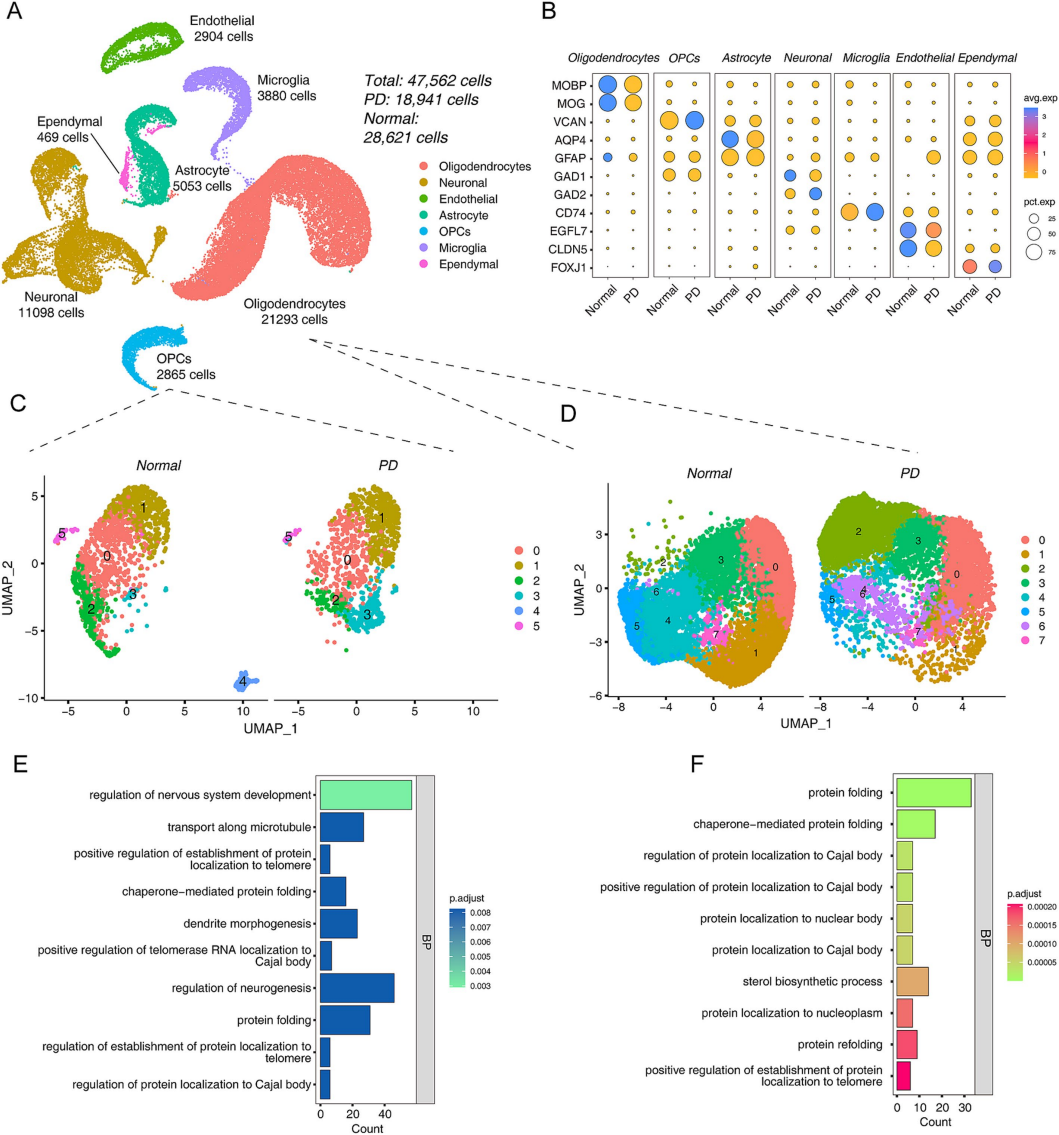
Integrated scRNA-seq data revealed a cellular landscape associated with PD. (A) The UMAP plots of control and PD cell distribution. (B) The dot plots show the expression patterns of different cell type marker genes in the PD and normal group. (C) UMAP plots of OPCs between PD and Normal group, left is normal and right is PD group. (D) UMAP plots of oligodendrocytes between PD and Normal group, left is normal and right is PD group. (E,F) The bar plot shows the top 10 GO functional annotations of DEGs of PD *vs* Normal group in OPCs (E) and oligodendrocytes (F).

Considering that the above analysis was performed in a rat model, to verify our hypothesis that oligodendrocyte abnormalities are involved in PD progression, we reanalyzed two human brain tissue scRNA-seq data sets (see detail in Supplementary Table S1). The UMAP map showed that 47,562 cells, including 28,621 cells in the normal group and 18,941 cells in the PD group, were obtained by dividing into 17 clusters, and a total of 7 cell types were annotated (Figure 3A; Supplementary Figure S3A). The dot plot showed the expression characteristics of related cell type marker genes in different groups, Oligodendrocytes [*MOBP*, *MOG* (Montague et al., 2006; Juryńczyk et al., 2019)], OPCs [*VCAN* (van Bruggen et al., 2017)], Astrocyte [*AQP4, GFAP* (Ikeshima-Kataoka, 2016; Jurga et al., 2021)], Neuronal [*GAD1, GAD2* (Kodama et al., 2012)], Microglia [*CD74* (Hwang et al., 2017)], Endothelial [*EGFL7, CLDN5* (Jang et al., 2011)] and Ependymal [*FOXJ1* (Shah et al., 2018)] (Figure 3B). Moreover, we used UMAP to display the maps of different groups and different samples (Supplementary Figures S3B,C).

Taking the WGCNA results into account, OPCs were also included in the analysis because they are precursors of oligodendrocytes. First, we extracted oligodendrocyte populations from the integrated data and re-clustered and visualized them. The results showed that there were significant differences in OPCs between the PD and Normal groups (Figure 3C). 1,435 DEGs were detected in OPCs (Supplementary Table S2). This result seems to suggest that PD progression was driven by OPCs. Interestingly, the top GO term of DEGs in OPCs was “regulation of nervous system development” (Figure 3E). The KEGG pathway results showed that “Chemical carcinogenesis - ROS” was most enriched (Supplementary Figure S3D). Next, the same analysis was performed on oligodendrocytes, and the UMAP showed the differences between PD and Normal groups (Figure 3D). Further, we performed differential analysis with a total of 851 DEGs (Supplementary Table S3). The GO annotation showed that the 851 DEGs were participated in “protein folding,” etc. (Figure 3F). The KEGG pathway results suggested that they were related to neurodegenerative diseases, such as “Parkinson disease,” “Huntington disease,” “Alzheimer disease” (Supplementary Figure S3E).

### 3.5 The pseudo-time trajectory of oligodendrocytes and OPCs

**Figure 4:**
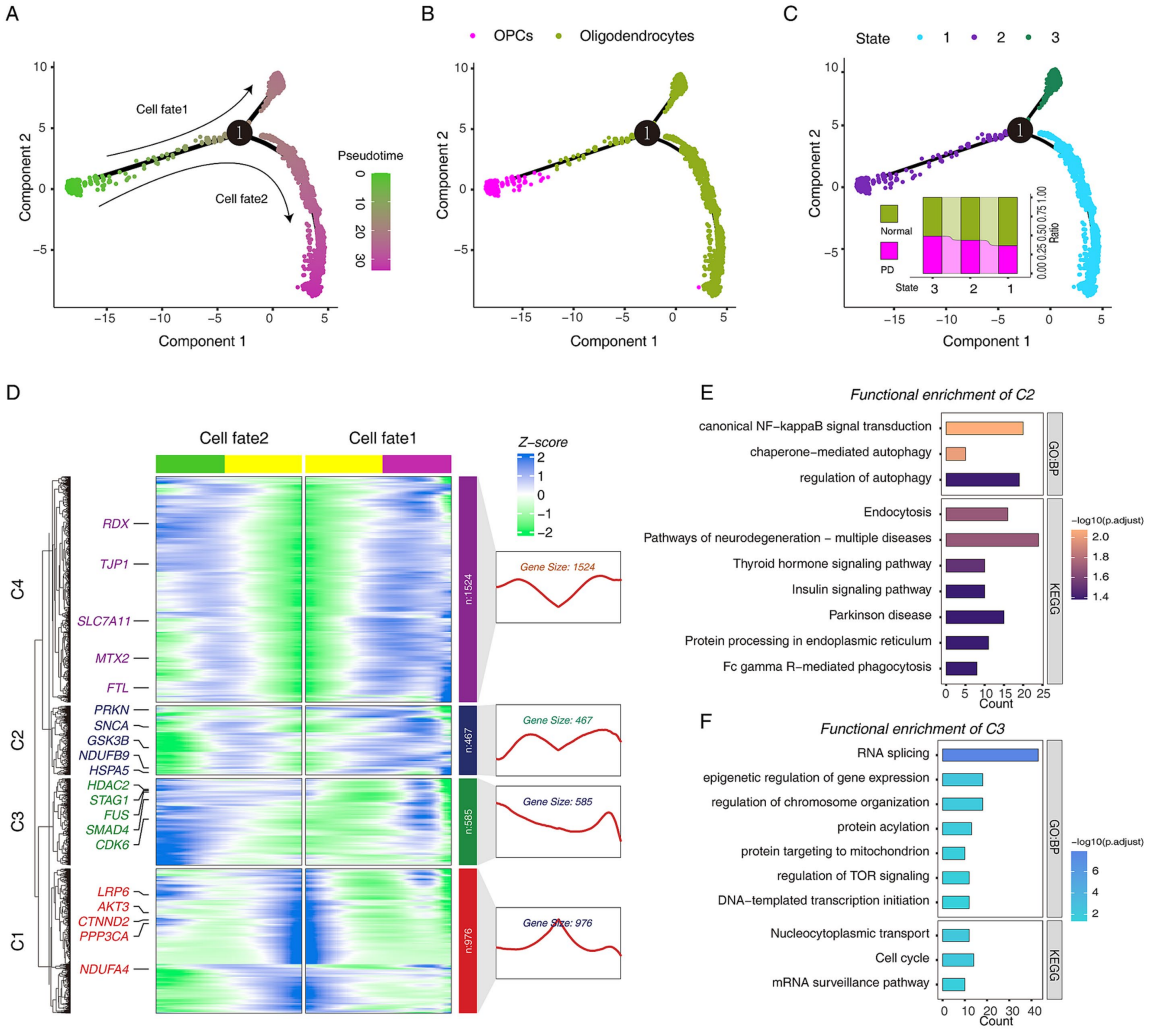
The pseudo-time trajectory analysis of oligodendrocytes and OPCs in Normal and PD. (A) Single-cell trajectories of oligodendrocytes and OPCs reveal distinct fate trajectories. (B) Single-cell trajectories of oligodendrocytes and OPCs along with celltype. (C) Single-cell trajectories of oligodendrocytes and OPCs during the three states through the pseudo-time. (D) The heatmap shows the dynamic changes in gene expression before and after the fate decision stage 1. (E) The bar plot shows the GO and KEGG pathway functional annotations of C2 cluster genes. (F) The bar plot shows the GO and KEGG pathway functional annotations of C3 cluster genes.

To dissect the fate decisions of OPCs/oligodendrocytes throughout the study period, they were sorted according to their gene expression patterns in a pseudo-temporal trajectory. We defined the stage in which OPCs were as the initial stage of the trajectory, and then a total of three cell stages were obtained, which were classified into two different cell fates (Figures 4A,B). Subsequently, we attempted to explore the transcriptional regulatory program of OPCs differentiation into oligodendrocytes, and the results suggested that PI3K/AKT/mTOR signaling may play a key role (Supplementary Figures S4A,B). Next, we observed the proportion of cells in different groups at three different cell stages, and we found that the proportion of PD cells was higher in state_3. We speculated that the stage where state_3 was located may be driven by the development of PD (Figure 4C). Next, we used the BAEM algorithm to try to parse the driving factors that lead to different cell fate decisions. Interestingly, we found that the emergence of cell fate_1 was driven by two distinct gene expression patterns. The *SNCA* gene was highly expressed in cell fate 1 and lowly expressed in cell fate_2, which is consistent with the fact that high *SNCA* expression promotes PD progression (Hentrich et al., 2020; Figures 4A,D). We divided the genes driving cell fate decisions into four gene sets. C2 was highly expressed in cell fate_1(PD), and functional enrichment analysis showed that “regulation of autophagy” and “Parkinson disease” were significantly enriched (Figure 4E). C3 was highly expressed in cell fate_2 (Normal), and functional enrichment analysis showed that “regulation of TOR signaling” and “cell cycle” were significantly enriched (Figure 4F).

### 3.6 Alterations in cell–cell communications between oligodendrocytes/OPCs and neuronal in PD

**Figure 5:**
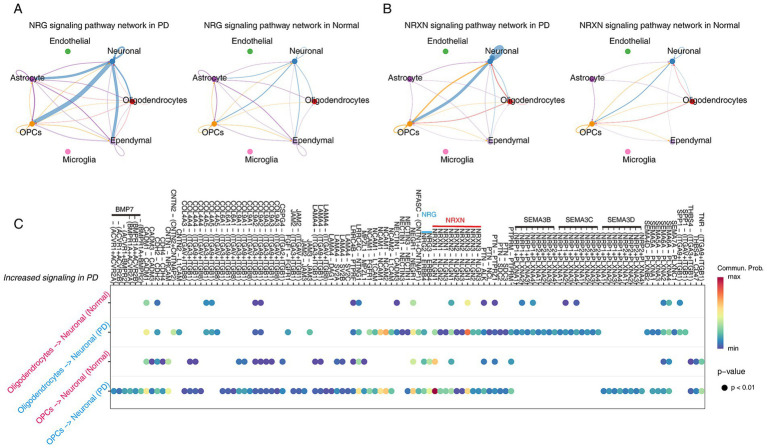
Inference of cell–cell communications in PD and Normal brain by CellChat. (A,B) Cellular communication in the NRG (A) and NRXN (B) signaling networks in PD and Normal. (C) The dot plots demonstrate the significantly increased cellular communication between OPCs-neuronal and oligodendrocytes-neuronal in PD.

To investigate the dynamics of cell–cell communications between oligodendrocytes/OPCs and neurons, we performed cell–cell communication analysis using CellChat (R software package v1.6.1). The results found that the number and intensity of cell communication in the PD group were higher than those in the normal group (Supplementary Figure S5A). Interestingly, cellular communication between oligodendrocytes/OPCs and neuronal cells was enhanced in PD group (Supplementary Figure S5B). Next, the signals of cell communication in different samples were aligned, and we found two signaling networks related to nervous system development have strong information flows, including NRG, NRGX signaling network (Supplementary Figure S5C). Interestingly, our cell communication networks observed that neurons communicated strongly with OPCs in both NRG and NRXN signaling (Figures 5A,B). Specifically, NRG3-ERBB4 was only present between OPC-neurons (Figure 5C). This result suggests that PD may be driven by OPCs because the communication between OPCs-neurons is closer than that between oligodendrocytes (Maldonado and Angulo, 2015; Xiao and Czopka, 2023; Boulanger and Messier, 2017; Orduz et al., 2015).

### 3.7 Constructing the gene co-expression network of OPCs/oligodendrocytes/neuronal at single-cell resolution

**Figure 6:**
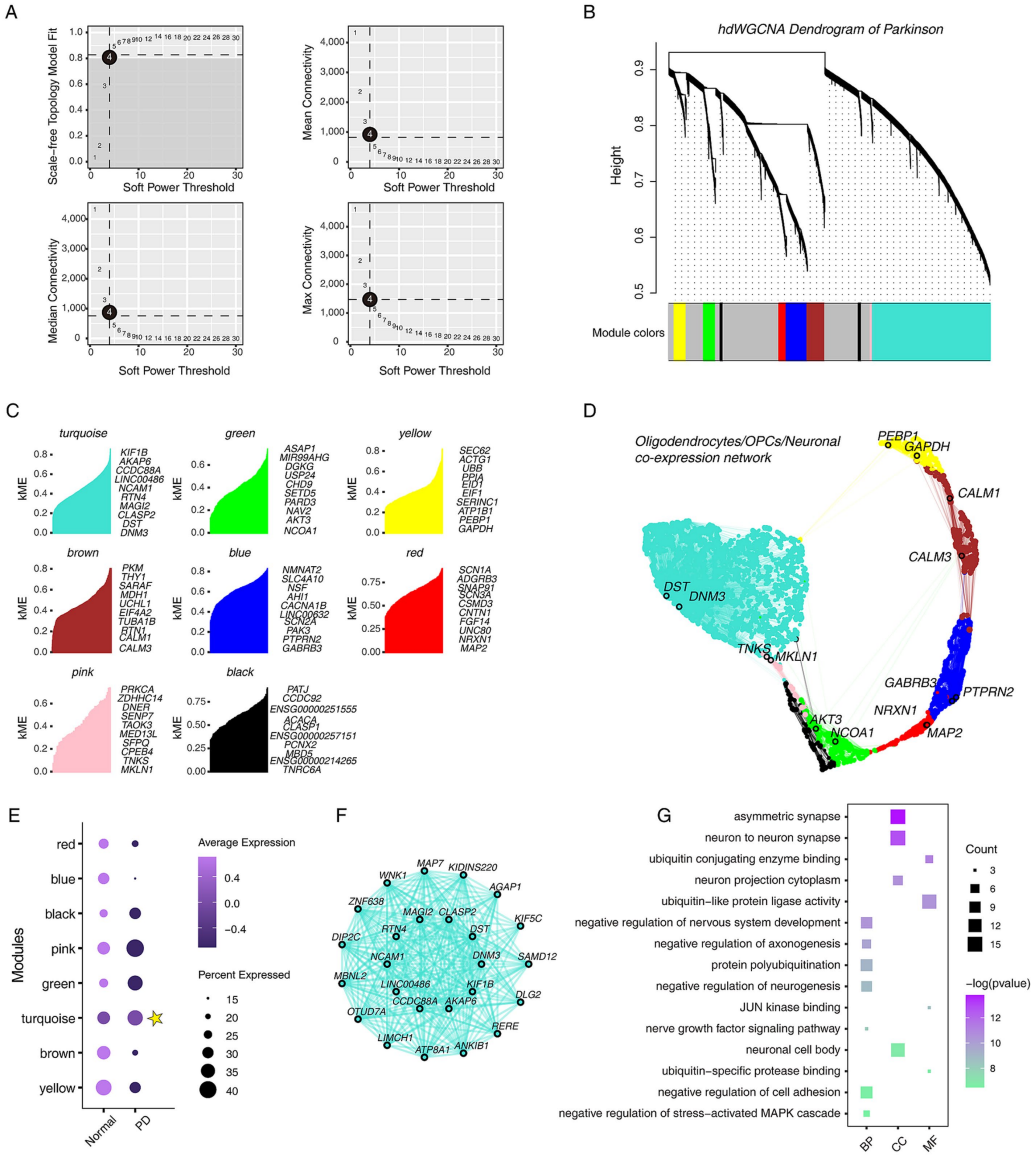
Overview of single-cell hdWGCNA results for oligodendrocytes, OPCs and neuronal cells. (A) Scale-free network topology analysis with different soft threshold powers in scale-free networks. The best soft threshold is highlighted. (B) The hierarchical clustering tree shows the relationship between genes and modules in the genes co-expression network. (C) Gene features within different modules, showing the top 10 genes with the highest connectivity. (D) The co-expression network of oligodendrocytes, OPCs and neuronal cells. (E) The dot plot shows the expression characteristics of different modules in different groups. Yellow star marks the modules with high expression in PD. (F) Co-expression network of the top 25 genes in the turquoise module. (G) The dot plot shows the GO functional annotations of top 200 genes in the turquoise module, including BP, CC, and MF.

To construct the gene co-expression network in OPCs/Oligodendrocytes/Neuronal and identify hub genes involved in PD, hdWGCNA analysis was performed. The hdWGCNA results suggested that when the soft threshold *β* = 4, scale-free network construction can be performed (Figure 6A). The hierarchical clustering tree showed the relationship between modules and genes (Figure 6B). A total of 8 valuable modules were identified (Figure 6C). The UMAP plot was used to display the OPCs/Oligodendrocytes/Neuronal gene co-expression network and highlighted the hub genes of each module involved in nervous system development (Figure 6D). The dot plot showed that the turquoise module is highly expressed in PD group, and the co-expression network of the top 25 hub genes was displayed (Figures 6E,F). Gene function annotation results showed that the hub genes of the turquoise module were involved in “negative regulation of neurogenesis,” “JNK kinase binding,” and so on (Figure 6G).

### 3.8 Identification of specific biomarkers for predicting PD progression

**Figure 7:**
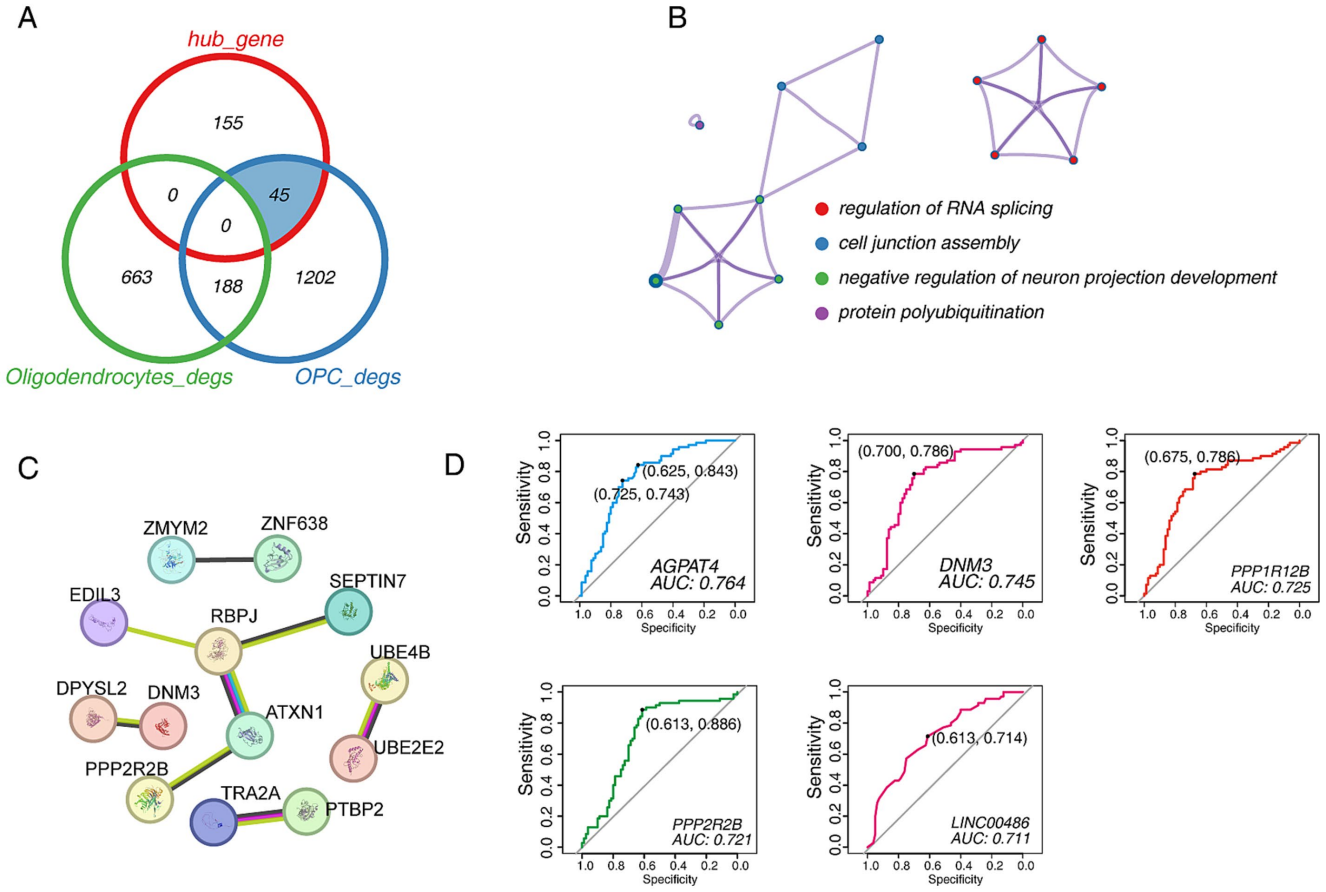
The identification of candidate biomarkers in PD. (A) Venn diagram shows the comparative analysis of hub genes and DEGs in the turquoise module. (B) The functional enrichment network of 45 candidate genes. (C) The PPI network of 45 candidate genes. (D) The ROC curve of candidate biomarkers. The figure shows the best sensitivity, specificity and AUC values.

We compared the relationship between hub genes and DEGs from scRNA-seq data, and the results showed that hub genes had 45 overlapping DEGs in OPCs groups. In contrast, it had no overlap with oligodendrocytes, which suggesting that PD progression may be more sensitive to OPCs. In other words, studying OPCs may be more critical (Figure 7A). Only four terms were enriched, including “regulation of RNA splicing,” “cell junction assembly,” “negative regulation of neuron projection development” and “protein polyubiquitination” (Figure 7B). Then, the PPI network was built, and showed PPP2R2B, DNM3 and ATXN1 may play a significant role (Figure 7C). Importantly, we sought to identify markers that drive PD and performed ROC binary analysis model in an independent dataset (Supplementary Table S1). The results showed that AGPAT4, DNM3, PPP1R12B, PPP2R2B and LINC00486 levels could distinguish PD patients from healthy controls. The area under the ROC curve was followed by 0.764, 0.745, 0.725, 0.721, and 0.711, which suggesting these five gene may serve as the potential biomarkers for predicting PD (Figure 7D).

## 4 Discussion

Emerging scRNA-seq applications have promoted the qualitative development of life sciences by shaping the unbiased transcriptomic roles of individual cells, such as mechanism of PD, reproductive development, identification of tumor subcellular populations, etc. (Smajić et al., 2022; Zhang et al., 2023a; Kieffer et al., 2020). With the optimization and development of analysis algorithms, it is easier to monitor transcriptional dynamics and construct cell development trajectories, and reveal the mechanisms of gene expression regulation (Morabito et al., 2023; Trapnell et al., 2014; Jin et al., 2021). Here, in this study, through bulk RNA-seq and single-cell WGCNA analysis, we highlighted the role of OPCs in PD progression. In addition, we identified five potential biomarkers using ROC binary analysis model, including AGPAT4, DNM3, PPP1R12B, PPP2R2B, and LINC00486.

By WGCNA analysis of bulk RNA-seq, the results suggested that oligodendrocyte transcriptional disorders may be involved in regulating PD progression. Oligodendrocyte is a type of glial cell whose primary function is to wrap around axons in the central nervous system and form an insulating myelin structure (Duncan et al., 2021). Its abnormality may induce neuronal damage (Pandey et al., 2022).

Next, we integrated two different single-cell data to provide more biological information. Cell trajectory analysis is able to infer the differentiation trajectory of cells during development or the evolution of cell subtypes (Trapnell et al., 2014). In our analysis, consistent with previous knowledge, OPCs, as precursor cells of oligodendrocytes (Huang et al., 2020), were in the early stages of the cell trajectory, while oligodendrocytes appear in two different cell fate stages along the cell trajectory, which can explain the results of WGCNA, that is, oligodendrocyte abnormalities promote PD. Then, the BEAM algorithm was used to analyze the transcriptional patterns that caused this cell fate transition and, as expected, found that two different transcriptional patterns drove the generation of cell fates. Significantly, we found that *SNCA* was in a state of gradually high expression in cell fates with a high proportion of PD cells. As far as we know, high expression of *SNCA* usually means the accumulation of *α*-Synuclein and the occurrence of PD (Hentrich et al., 2020; Oliveira et al., 2015). Cell communication analysis can provide differential information about how cell subpopulations within different groups achieve signal transduction through ligand-receptor binding (Jin et al., 2021). The analysis results showed that the inflammatory signaling network is significantly enhanced in PD group, such as IL16 and CD22. Because it was found that the neural response to inflammation in the brain area of PD patients was enhanced (Pereira et al., 2023). Moreover, a large number of ligand-receptor interactions were observed to be enhanced in OPCs-neuronal and oligodendrocytes-neuronal in PD. Interestingly, we found that OPCs-neuronal signaling was stronger than oligodendrocytes-neuronal signaling. One example is the enhancement of NRG ligand-mediated signaling, which was highlighted in a review regarding its relevance to PD (Iwakura and Nawa, 2013). The above results supported that changes in the transcriptional patterns of oligodendrocytes/OPCs are involved in regulating the progression of PD.

On the other hand, we explored the gene co-expression network of oligodendrocytes/OPCs/neuronal cells at the single-cell level by hdWGCNA analysis, a novel and soon-to-be widely used algorithm (Morabito et al., 2023; Sziraki et al., 2023). A new functional module involved in regulating PD was identified, and the hub genes showed the presence of genes that potentially regulate the development of the nervous system, such as *DNM3* and *DST* (Trinh et al., 2016; Lalonde and Strazielle, 2023). Furthermore, we compared and analyzed the hub genes with DEGs. Interestingly, the hub genes overlapped only with the DEGs of OPCs, but not with those of oligodendrocytes, which seems to indicate that abnormal developmental regulation of OPCs drives PD formation. A recent report showed that the transcriptional dynamics of OPCs can effectively predict the clinical prognosis of PD (Dehestani et al., 2023). The above suggested that more work is needed to focus on the mechanisms by which OPCs regulate the progression of PD. Importantly, we attempted to use the ROC binary analysis model to identify effective biomarkers to identify PD better. Based on the AUC values, we selected five biomarkers, including AGPAT4, DNM3, PPP1R12B, PPP2R2B, and LINC00486. AGPAT4 is mainly involved in regulating lipid metabolism and is an acylglycerol phosphate acyltransferase. It has been reported to be abnormally expressed in PD (Girard et al., 2020). DNM3 belongs to the dynein family and is involved in regulating the development of the nervous system (GO database), but its role in PD seems to be ambiguous (Trinh et al., 2016; Berge-Seidl et al., 2019). PPP1R12B encodes protein phosphatase 1 regulatory subunit 12B, which may be involved in regulating PD through LRRK2 (Häbig et al., 2008). Another evidence showed that it interacts with IL16, and in our analysis, the IL16 signaling network was significantly enhanced in PD (Bannert et al., 2003), which further emphasized that PPP1R12B plays a key role in PD. PPP2R22B encodes the Serine/threonine-protein phosphatase 2A 55 kDa regulatory subunit B beta isoform, which is also a protein phosphatase involved in the negative control of cell growth and division, and has been reported to be downregulated in PD (Mayer et al., 1991; Cheng et al., 2009; Kim et al., 2017). LINC00486 is a long intergenic non-protein coding RNA that participates in “Protein aggregates” through epigenetic regulation (Shmookler Reis et al., 2021). It is worth noting that Lewy bodies formed by the accumulation of *α*-Synuclein are a prominent pathological feature of PD (Dong-Chen et al., 2023; Hentrich et al., 2020), although there is no direct evidence showing the relationship between LINC00486 and Lewy bodies.

Although we used the latest algorithms and described the transcriptional signatures of OPCs/oligodendrocytes/neuronal cells under normal and PD conditions, our analysis is not without limitations. The first and most prominent limitation is that we lack the necessary experimental validation, although these data are derived from human samples of PD disease. Our analysis showed that these candidate biomarkers are DEGs that were highly associated with PD, but experimental validation is still lacking. In addition, we have not explored the molecular mechanisms by which OPCs participate in regulating PD, which needs to be strengthened in future studies.

## 5 Conclusion

In summary, our work highlights the potential value of OPCs in driving PD and screens five potential biomarkers, including AGPAT4, DNM3, PPP1R12B, PPP2R2B, and LINC00486.

## Data Availability

The original contributions presented in the study are included in the article/Supplementary material, further inquiries can be directed to the corresponding authors.

## Footnotes

^1^https://metascape.org/gp/index.html#/main/step1

^2^https://string-db.org/, v 12.0.

## Supplementary material

SUPPLEMENTARY FIGURE S1 Transcriptome differences in PD rat models. (A) The volcano plot shows the DEGs from PD_young *vs* young group. The horizontal axis is log2 FoldChang, and the vertical axis is −log10(*padj*). (B) The top 10 GO terms of DEGs in (A), the color represents padj, and the horizontal axis expresses the number of genes. (C) The top 10 KEGG pathways of DEGs in (A), the color represents padj, and the size of dot expresses the number of genes. (D) The volcano plot shows the DEGs from PD_old *vs* old group. The horizontal axis is log2 FoldChang, and the vertical axis is −log10(*padj*). (E) The top 10 GO terms of DEGs in (D), the color represents *padj*, and the horizontal axis expresses the number of genes.

SUPPLEMENTARY FIGURE S2 (A) The hierarchical clustering tree shows the discreteness of all samples.

SUPPLEMENTARY FIGURE S3 (A,B) The UMAP plots shows the seurat_clusters, groups (B left) and smaples (B right) in integrated data (GSE140231 and GSE157783). (C) The UMAP plots shows the groups (up) and smaples (down) in OPCs. (D) The UMAP plots shows the groups (up) and smaples (down) in oligodendrocytes. (E) The dot plot shows all KEGG pathways of DEGs of PD *vs* Normal group in OPCs. (F) The dot plot shows the top 10 KEGG pathways of DEGs of PD *vs* Normal group in oligodendrocytes.

SUPPLEMENTARY FIGURE S4 (A) Expression trends of VCAN, MOBP, and MOG in pseudo-time trajectories. (B) Heatmap shows the changes in the expression of regulatory genes in the differentiation of OPCs into oligodendrocytes. (C) The dot plot shows the KEGG pathway functional annotations of regulatory genes in the differentiation of OPCs into oligodendrocytes.

SUPPLEMENTARY FIGURE S5 (A) The bar graph shows the cell communication profile in brain tissues of PD and Normal group, with the number of cell communications on the left and the intensity of cell communication on the right. (B) The network diagram shows the amount of cell communication in different cell types, with the PD group on the left and the Normal group on the right. (C) The bar chart shows the interaction network between PD and Normal samples arranged by the overall information flow differences.

SUPPLEMENTARY TABLE S1 The detail information of data collection.

SUPPLEMENTARY TABLE S2 The DEGs of oligodendrocytes between PD vs. Normal group.

SUPPLEMENTARY TABLE S3 The DEGs of OPCs between PD vs. Normal group.
