# Supplementary figures and images for: Identification of biomarkers in Parkinson’s disease by comparative transcriptome analysis and WGCNA highlights the role of oligodendrocyte precursor cells
Source: Front Aging Neurosci. 2024 Nov 20;16:1485722. doi: 10.3389/fnagi.2024.1485722 (PMC11615075; doi:10.3389/fnagi.2024.1485722)

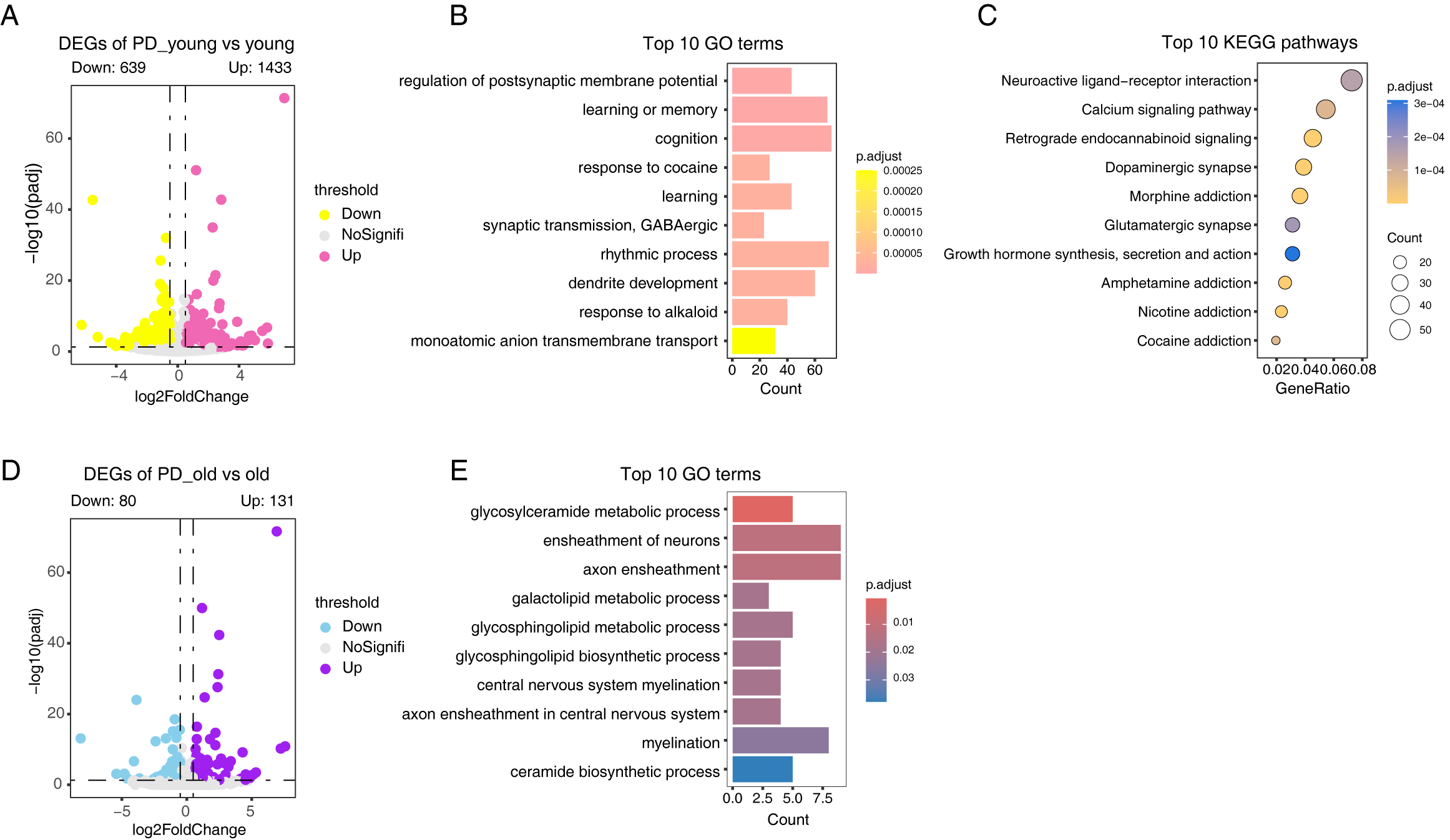

Supplement: SUPPLEMENTARY FIGURE S1 — Transcriptome differences in PD rat models. (A) The volcano plot shows the DEGs from PD_young vs young group. The horizontal axis is log2 FoldChang, and the vertical axis is −log10(padj). (B) The top 10 GO terms of DEGs in (A), the color represents padj, and the horizontal axis expresses the number of genes. (C) The top 10 KEGG pathways of DEGs in (A), the color represents padj, and the size of dot expresses the number of genes. (D) The volcano plot shows the DEGs from PD_old vs old group. The horizontal axis is log2 FoldChang, and the vertical axis is −log10(padj). (E) The top 10 GO terms of DEGs in (D), the color represents padj, and the horizontal axis expresses the number of genes. [file Image_1.tif]

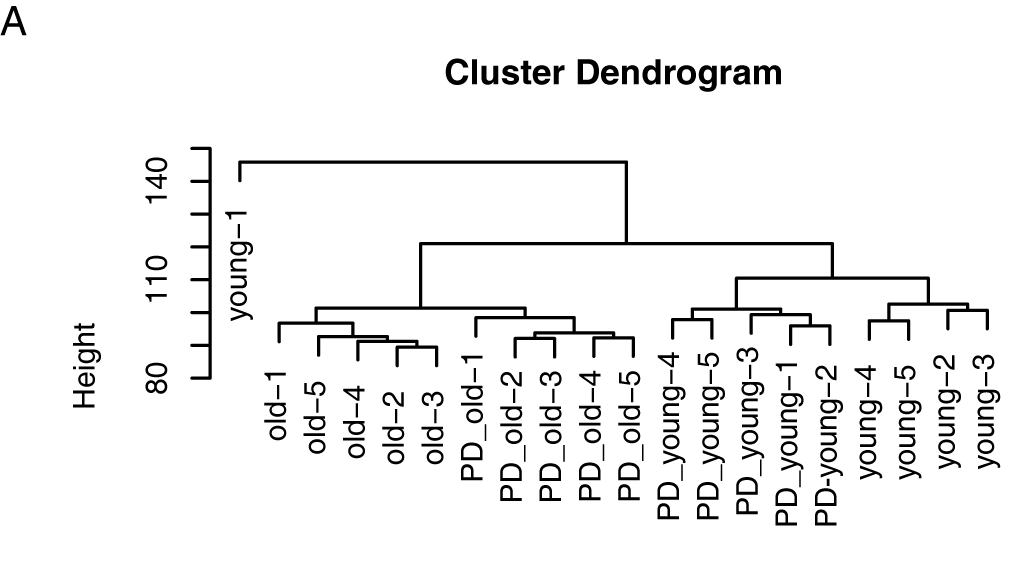

Supplement: SUPPLEMENTARY FIGURE S2 — (A) The hierarchical clustering tree shows the discreteness of all samples. [file Image_2.tif]

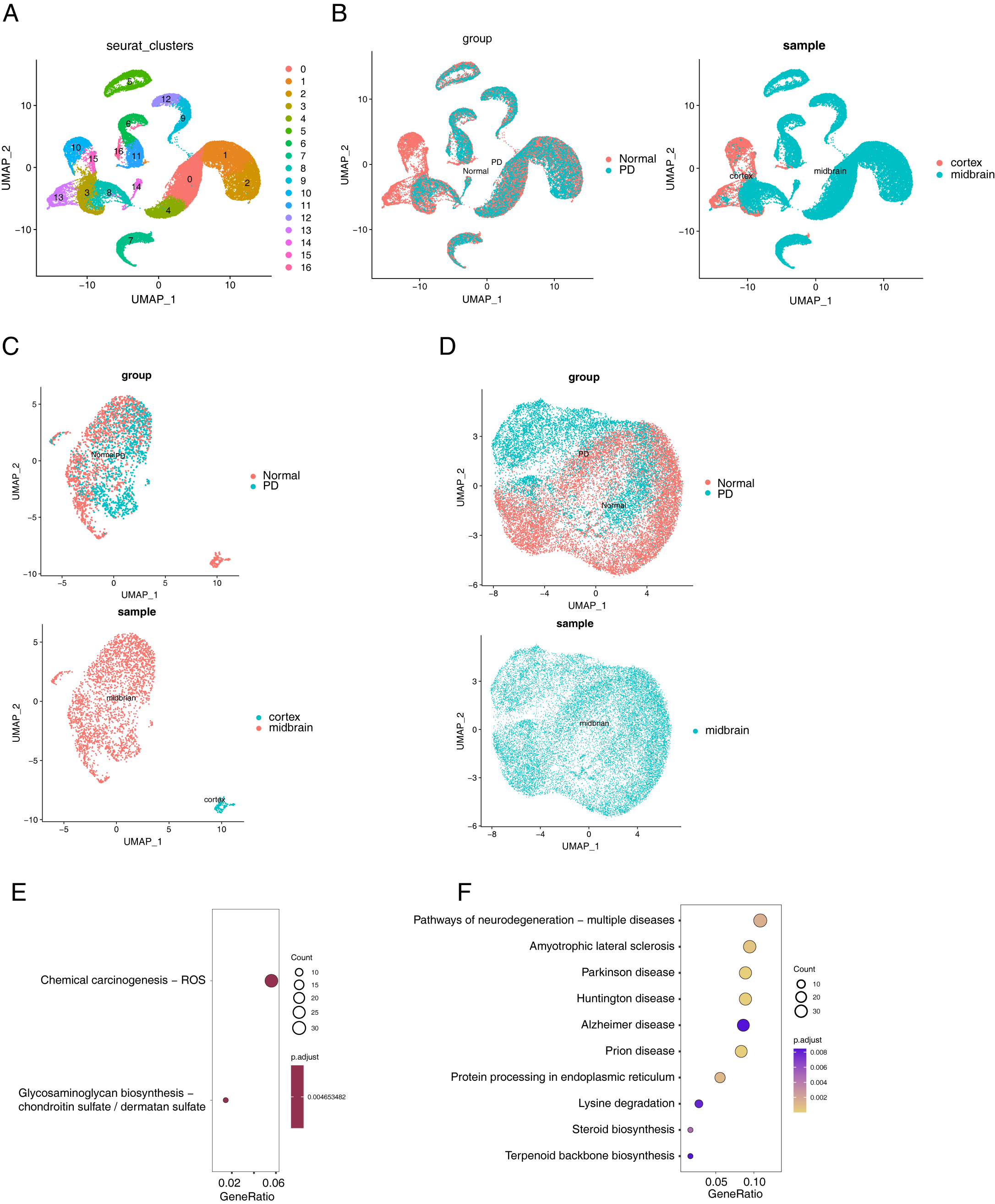

Supplement: SUPPLEMENTARY FIGURE S3 — (A,B) The UMAP plots shows the seurat_clusters, groups (B left) and smaples (B right) in integrated data (GSE140231 and GSE157783). (C) The UMAP plots shows the groups (up) and smaples (down) in OPCs. (D) The UMAP plots shows the groups (up) and smaples (down) in oligodendrocytes. (E) The dot plot shows all KEGG pathways of DEGs of PD vs Normal group in OPCs. (F) The dot plot shows the top 10 KEGG pathways of DEGs of PD vs Normal group in oligodendrocytes. [file Image_3.tif]

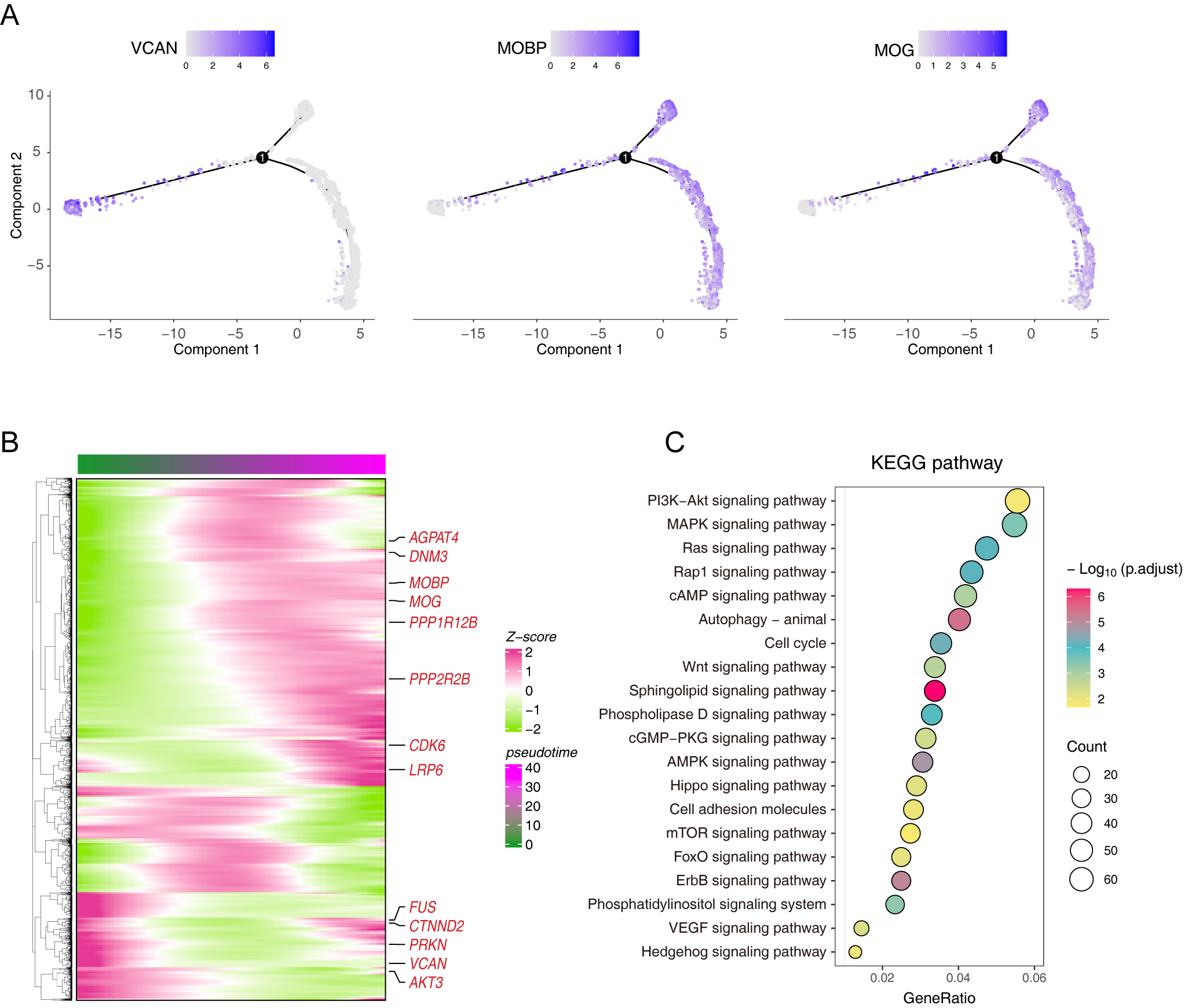

Supplement: SUPPLEMENTARY FIGURE S4 — (A) Expression trends of VCAN, MOBP, and MOG in pseudo-time trajectories. (B) Heatmap shows the changes in the expression of regulatory genes in the differentiation of OPCs into oligodendrocytes. (C) The dot plot shows the KEGG pathway functional annotations of regulatory genes in the differentiation of OPCs into oligodendrocytes. [file Image_4.tif]

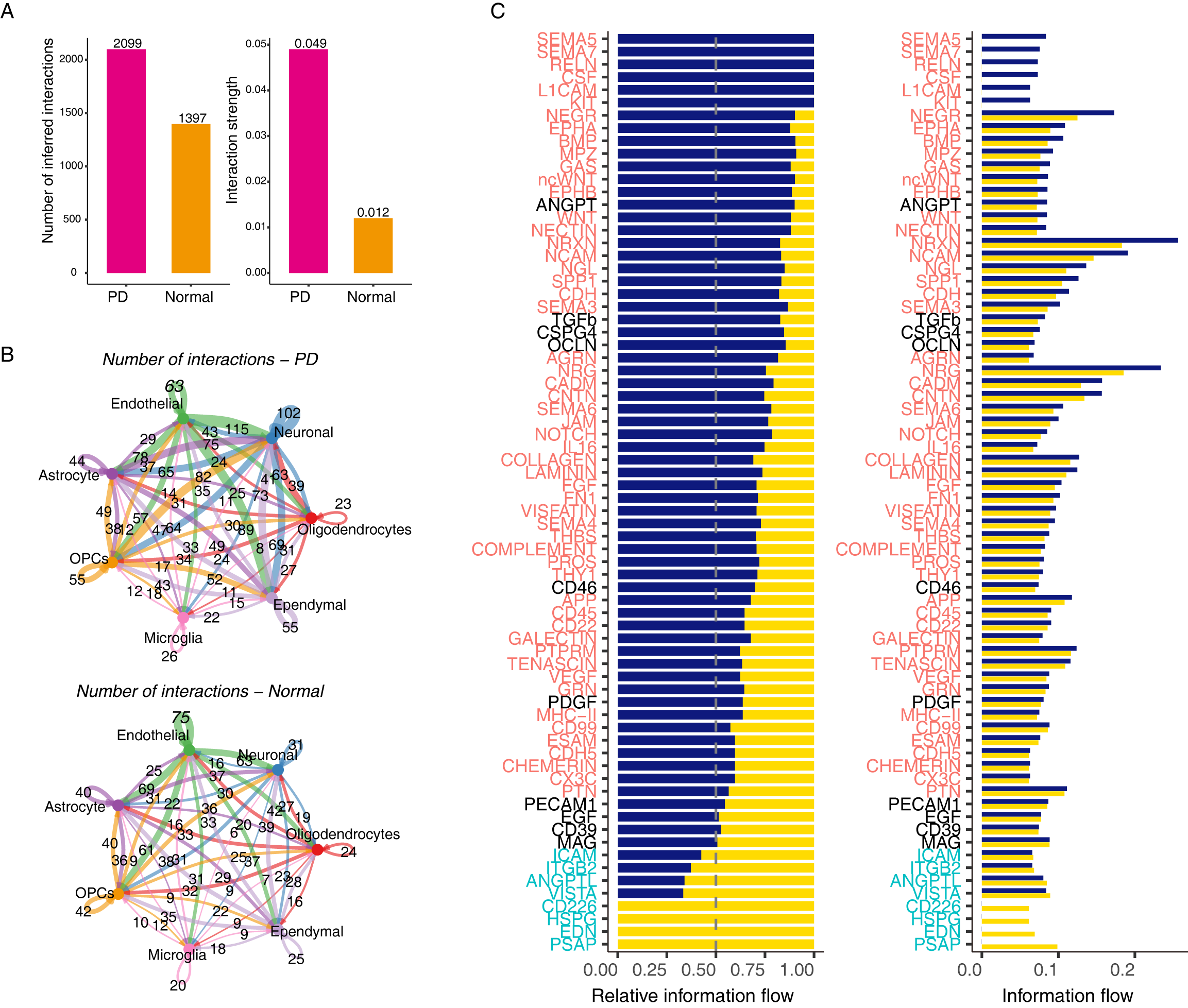

Supplement: SUPPLEMENTARY FIGURE S5 — (A) The bar graph shows the cell communication profile in brain tissues of PD and Normal group, with the number of cell communications on the left and the intensity of cell communication on the right. (B) The network diagram shows the amount of cell communication in different cell types, with the PD group on the left and the Normal group on the right. (C) The bar chart shows the interaction network between PD and Normal samples arranged by the overall information flow differences. [file Image_5.tif]
