# Supplementary material for: Identification of biomarkers in Parkinson’s disease by comparative transcriptome analysis and WGCNA highlights the role of oligodendrocyte precursor cells
Source: Front Aging Neurosci. 2024 Nov 20;16:1485722. doi: 10.3389/fnagi.2024.1485722 (PMC11615075; doi:10.3389/fnagi.2024.1485722)
Supplement: SUPPLEMENTARY TABLE S1 — The detail information of data collection. [file Table_1.docx]

**Table S1, The detail information of data collection.**

| **GEO ID** | **Dataset Type** | **Organism** | **Main Analysis** | **Related Figures** |
| --- | --- | --- | --- | --- |
| GSE150646 | Bulk RNA-seq | Rat,  frontal cortex | - WGCNA - Identification DEGs | Fig1,2,S1,S2 |
| GSE140231 | Single-cell RNA-seq | Human,  cortex | - hdWGCNA - cell–cell communications - pseudo-time trajectories | Fig3,4,5,6,S3,S4,S5 |
| GSE157783 | Single-cell RNA-seq | Human,  midbrain | - hdWGCNA - cell–cell communications - pseudo-time trajectories | Fig3,4,5,6,S3,S4,S5 |
| GSE205450 | Bulk RNA-seq | Human,  Striatum | - Construction of ROC binary analysis model | Fig7 |

**For single-cell detail information.**

The number of different cell types detected in different sample

| **Celltype** | **Cortex** (GSE140231) | **Midbrain** (GSE157783) |
| --- | --- | --- |
| Oligodendrocytes | 0 | 21293 |
| Neuronal | 5823 | 5275 |
| Endothelial | 0 | 2904 |
| Astrocyte | 310 | 4743 |
| OPCs | 114 | 2751 |
| Microglia | 2 | 3878 |
| Ependymal | 3 | 466 |

The number of different cell types detected in different group

| **Celltype** | **Normal** | **PD** |
| --- | --- | --- |
| Oligodendrocytes | 12777 | 8516 |
| Neuronal | 8735 | 2363 |
| Endothelial | 1635 | 1269 |
| Astrocyte | 2450 | 2603 |
| OPCs | 1652 | 1213 |
| Microglia | 1186 | 2694 |
| Ependymal | 186 | 283 |
